# Peroral plication of the esophagus for end-stage achalasia with sump formation after peroral endoscopic myotomy

**DOI:** 10.1055/a-2954-0449

**Published:** 2026-10-05

**Authors:** Tommy Rizkala, Meddy Dalex, Marie Philippart, François Kungu, Jeanine Wakim El-Khoury, Domenico Galasso, Sébastien Godat

**Affiliations:** 1 Department of Gastroenterology and Hepatology 30635 Lausanne University HospitalLausanneVDSwitzerland

Achalasia is characterized by failure of the lower esophageal sphincter (LES) relaxation and absent peristalsis. 1​ A minority of patients progress to end-stage disease, with a dilated, sigmoid “megaesophagus” and a dependent “sump” that retains food and secretions despite an adequately treated LES, causing disabling regurgitation and aspiration. 1​ ​2​ Esophagectomy is the traditional rescue but carries high morbidity. Peroral plication of the esophagus (POPE), or endoscopic esophagoplasty, applies the suturing technique used in endoscopic sleeve gastroplasty to reduce the sump and straighten the esophagus, restoring gravity-dependent emptying. 2​ ​3​

A 66-year-old woman with type 1 achalasia underwent peroral endoscopic myotomy (POEM) in 2019 aborted for technical failure due to the sigmoid configuration of the distal esophagus, and a completed redo-POEM in 2022. She had persistent dysphagia and disabling food regurgitation. Work-up with gastroscopy, EndoFLIP, and barium esophagram showed a massive, longstanding distension of the distal esophagus with a sigmoid appearance forming a dependent “sump.” The lower esophageal sphincter opened well, confirmed by EndoFLIP measurements within normal limits (distensibility index >9). The findings represent end-stage decompensation of longstanding achalasia: the dilated esophagus acts as an intrathoracic reservoir that cannot empty despite a patent sphincter. The obstacle was therefore the redundant reservoir, not the sphincter.

Having declined esophagectomy, she underwent POPE ( Video 1 ), to reduce the sump and straighten the esophagus. POPE was performed under general anesthesia, endoscopy showed a patulous cardia (a lower esophageal sphincter of 40 cm from the incisors) with a large food residue. First, the retained food was aspirated and cleared ( Fig. 1 ). Second, boundary marking to guide the suturing was performed with argon plasma coagulation dots. Third, esophagoplasty was performed with an Apollo OverStitch endoscopic suturing system on a double-channel gastroscope: full-thickness plication sutures over the distal 13 cm of the esophagus reduced the lumen by three-quarters ( Fig. 2 ). Tubularization was complete and the stomach was desufflated ( Fig. 3 ). The patient was discharged after a short stay. At 2 months follow-up, she reported marked symptomatic improvement; surveillance endoscopy showed a far less dilated esophagus, with the plication sutures in place but partial dehiscence of the distal suture line and minor residual food. Clinical follow-up was arranged.

**Video 1** Peroral plication of the esophagus: endoscopic esophagoplasty by suture plication of a sigmoid megaesophagus in end-stage achalasia after POEM.


**Fig. 1:**
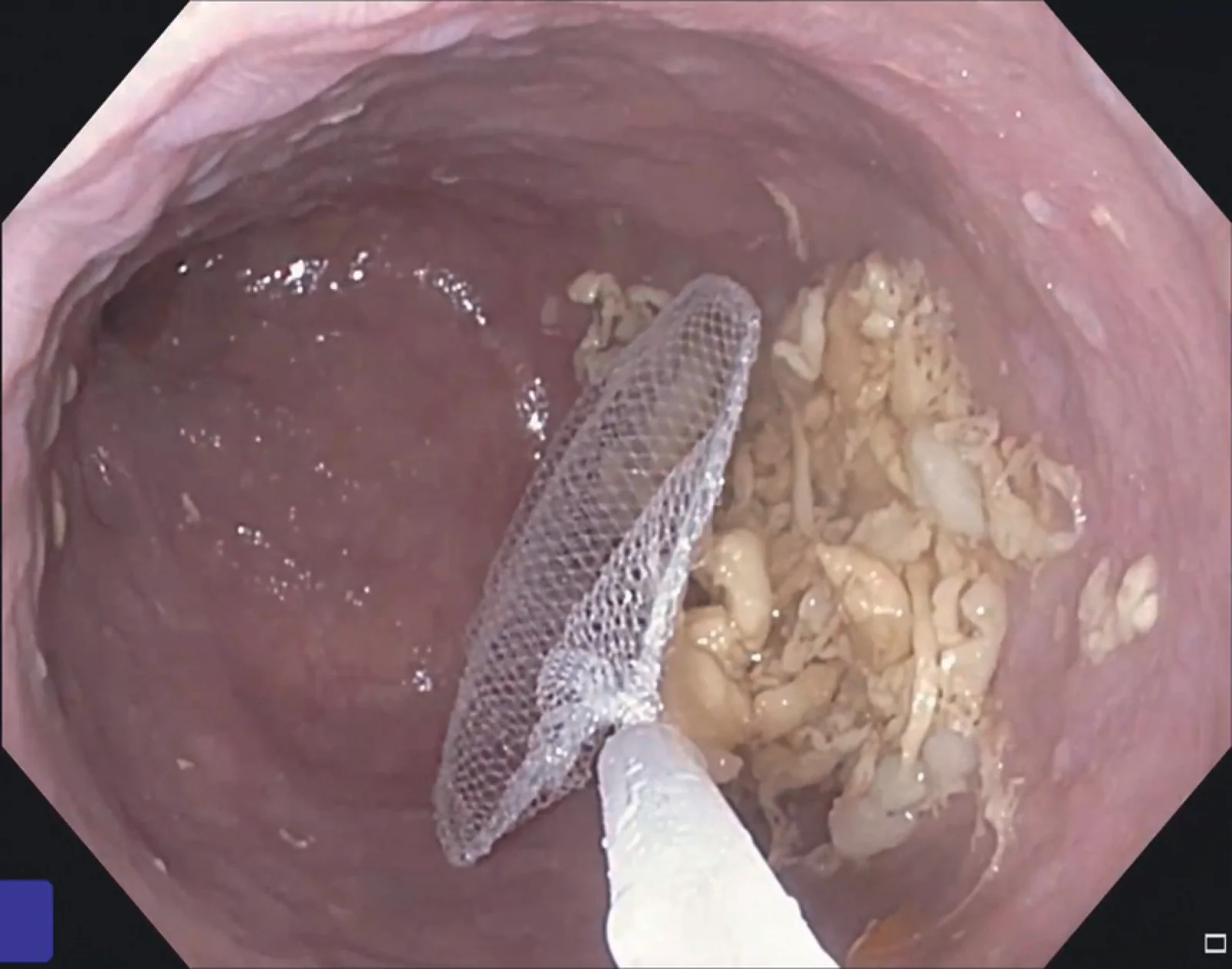
Pre-procedural endoscopy: dilated distal esophagus with a large volume of retained food and a patulous cardia.

**Fig. 2:**
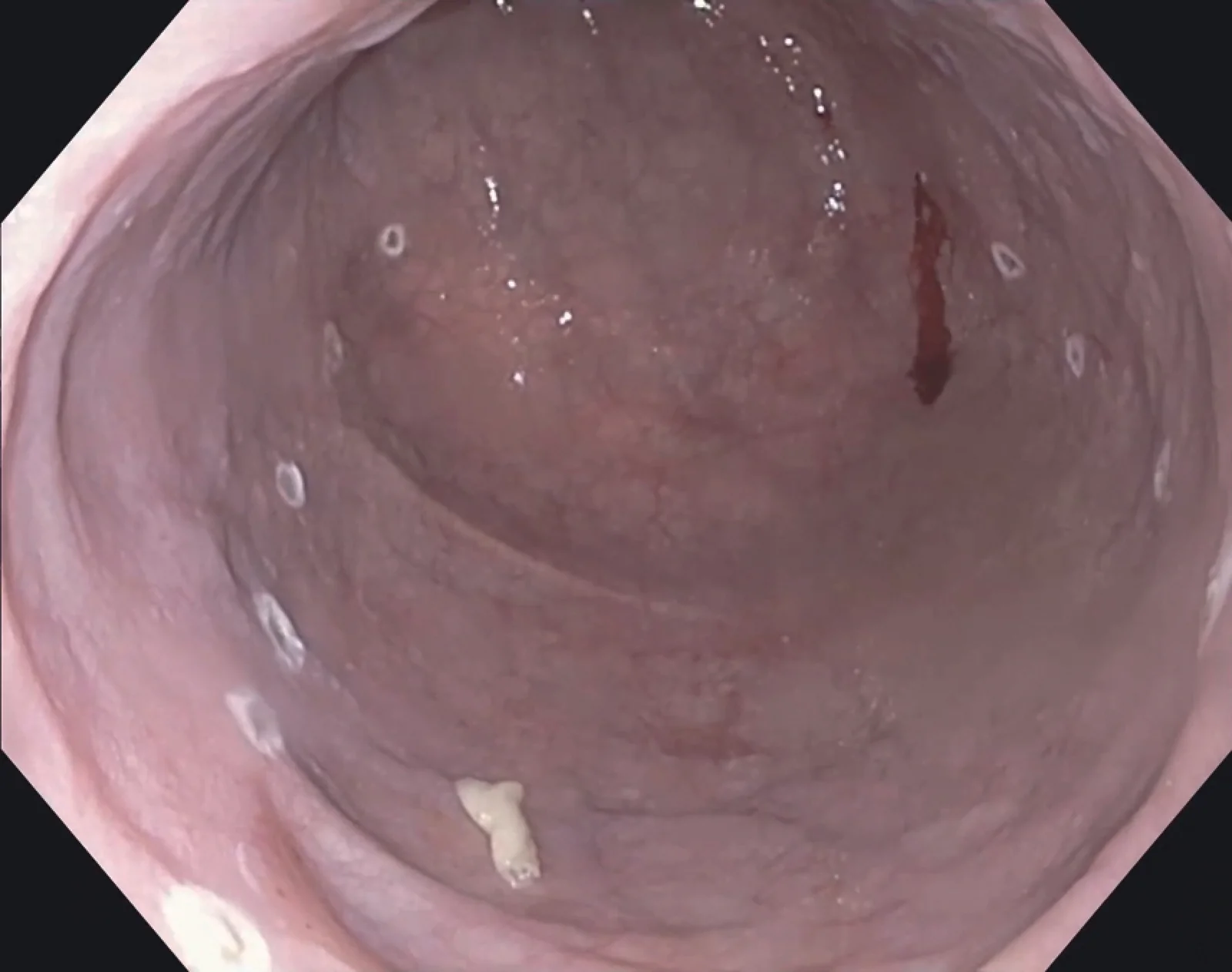
Argon plasma coagulation dots boundary marking to guide the suturing.

**Fig. 3:**
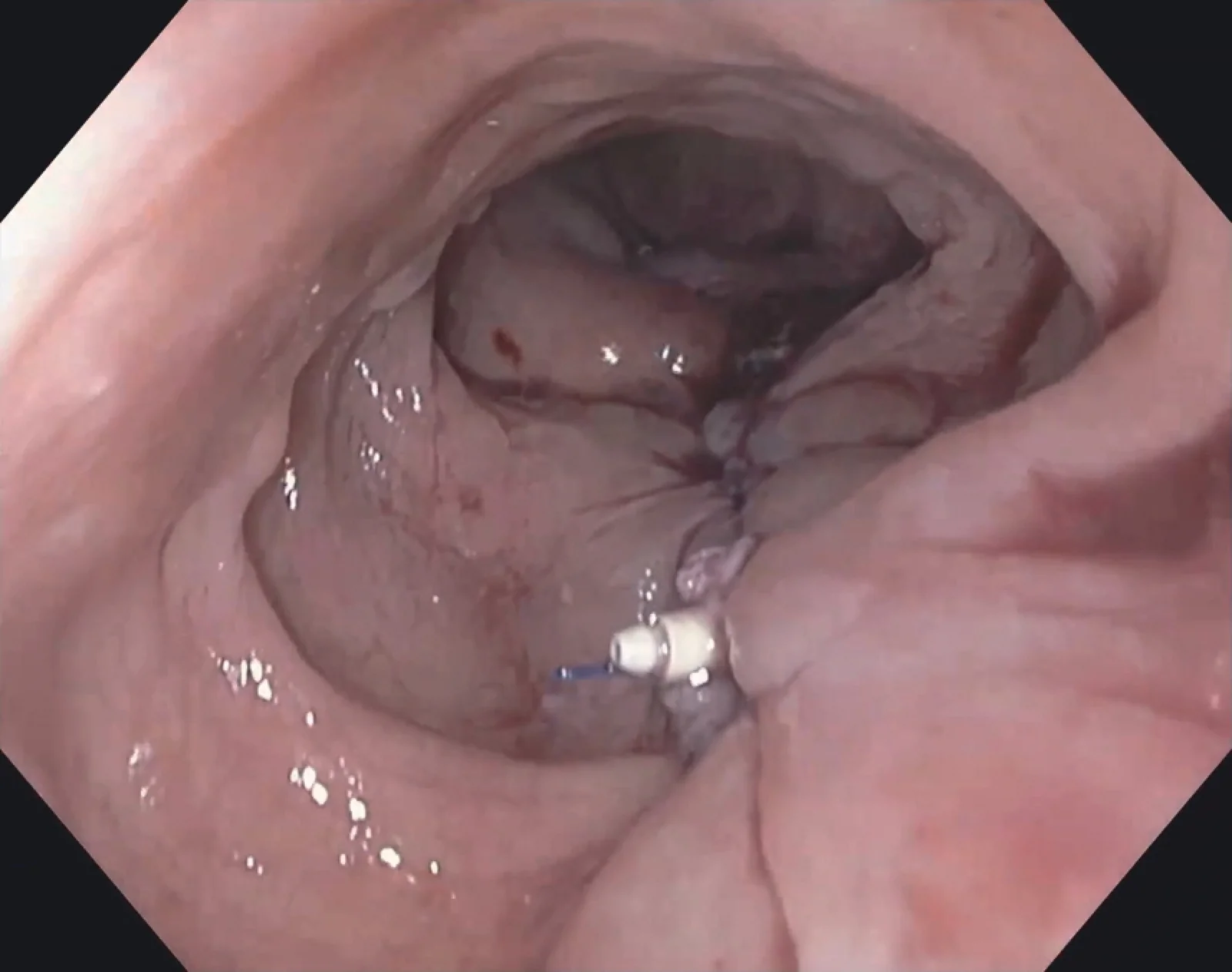
Esophagoplasty: full-thickness plication sutures placed with an Apollo OverStitch system.

POPE may offer a minimally invasive, repeatable alternative to esophagectomy in end-stage achalasia with sump formation when the LES is already patent, including after POEM and redo-POEM 3​ ​4​ , plication durability warrants further study.

Endoscopy_UCTN_Code_TTT_1AO_2AJ

## Data Availability

No new data were generated or analyzed for this article.

## Declaration of GenAI Use

The authors confirm that no generative artificial intelligence was used in the preparation of this manuscript.
